# The relationship between nursing students’ digital literacy and their levels of clinical decision-making

**DOI:** 10.3389/fpubh.2026.1948572

**Published:** 2026-09-17

**Authors:** Figen Çalışkan

**Affiliations:** Nursing Education, Nursing Department, Health Science Faculty, Trakya University, Edirne, Türkiye

**Keywords:** clinical decision-making, digital competence, digital literacy, nursing education, nursing students

## Abstract

**Background:**

The rapid integration of digital health technologies into healthcare has made digital literacy a fundamental competency for nursing students. Digital literacy may facilitate evidence-based practice and enhance clinical decision-making by supporting the effective and critical use of digital health technologies. However, evidence regarding the relationship between digital literacy and clinical decision-making among nursing students remains limited. Therefore, this study aimed to examine the relationship between nursing students’ digital literacy levels and clinical decision-making skills.

**Methods:**

This was a descriptive, cross-sectional, and correlational study. A total of 271 nursing students participated in the study. Data were collected between June 2, and June 19, 2026, using an Information Form, the Digital Literacy Scale, and the Clinical Decision-Making in Nursing Scale. Data were analyzed using descriptive statistics, Student’s t-test, one-way ANOVA, Mann–Whitney U test, Pearson correlation analysis, and multiple linear regression analysis.

**Results:**

The Digital Literacy Scale mean score was 65.40 ± 10.62, whereas the Clinical Decision-Making in Nursing Scale mean score was 137.49 ± 14.04. Digital literacy scores differed significantly according to gender, academic year, daily internet use, and purpose of internet use (all *p* < 0.05). Clinical decision-making scores differed significantly by gender, daily internet use, and health information-seeking behavior (all *p* < 0.05). Digital literacy was positively associated with clinical decision-making, with weak-to-moderate correlations. Multiple linear regression analysis showed that only the Attitude subscale of digital literacy significantly predicted clinical decision-making, explaining 12.6% of the variance (*β* = 0.344, *p* = 0.001).

**Conclusion:**

Digital literacy was positively associated with clinical decision-making among nursing students, and the attitude subscale of digital literacy emerged as a significant predictor of clinical decision-making. These results highlight the importance of fostering digital literacy, particularly positive attitudes toward digital technologies, within undergraduate nursing education to better prepare future nurses for digitally supported healthcare environments.

## 1 Introduction

Digital transformation has led to changes in the delivery of health care, just as it has in many other areas of life. Numerous digital technologies—such as electronic health record systems, clinical decision support systems, telemedicine, telehealth, mobile health applications, and artificial intelligence-supported systems—have been integrated into health care services. The integration of these technologies into healthcare has increased the efficiency of care processes, improved clinical workflows, enhanced the quality of care, and contributed to cost-effectiveness (1, 2). At the same time, the widespread use of digital health technologies in healthcare has made it necessary for nurses to possess not only clinical knowledge and skills but also digital literacy skills that enable them to use these digital technologies effectively, safely, and critically.

Digital literacy can be defined as an individual’s ability to effectively use digital tools and technologies to access information, evaluate information, generate new information, and appropriately share the information they have generated (3). Nurses with digital literacy skills are able to integrate digital health technologies more effectively into clinical practice and manage care processes more safely and efficiently. These skills support the effective use of digital health technologies; it has been reported that the effective use of these technologies strengthens communication with patients, reduces clinical errors and workload, and improves time management (4, 5). Therefore, digital literacy emerges as an integral component of the educational process to ensure that nursing students graduate with these skills (6). Throughout their undergraduate education, nursing students encounter e-learning platforms, digital educational materials, simulation applications, electronic health record systems, and various digital clinical learning environments (7). In addition, access to current evidence in clinical practice, the management of patient information, and the use of digital health technologies also necessitate that students demonstrate a certain level of digital competence (8). Therefore, developing nursing students’ digital literacy skills is important not only for academic success but also for their ability to provide safe, effective, and evidence-based care and to acquire effective clinical decision-making skills.

Decision-making is an integral part of the nursing process (9). Clinical decision-making is practiced daily to enable nurses to provide effective and safe care to patients (10). Clinical decision-making is a complex process that involves the nurse observing the patient’s condition, applying nursing knowledge and experience based on critical thinking, and carefully evaluating patient data (11). Clinical decision-making is a process that involves nurses’ skills in critical thinking, clinical reasoning, problem-solving, and judgment (12). The decisions nurses make affect patient safety, patient care, and patient outcomes (13, 14). Due to the increasing complexity of care in healthcare services and the widespread integration of digital health technologies into clinical practice, the need for nurses to make timely, rapid, accurate, and evidence-based clinical decisions has become even more critical (15). For this reason, developing nursing students’ clinical decision-making skills is considered one of the fundamental goals of nursing education; it is recommended that these skills be incorporated into both undergraduate and continuing education programs to ensure students can provide safe and high-quality care (13, 16). In this context, elucidating the relationship between digital literacy and clinical decision-making among nursing students is important for the advancement of nursing education and the effective integration of digital competencies into educational programs.

In recent years, digital literacy and clinical decision-making skills have emerged as important research topics in nursing education. However, in the current literature, these two concepts have mostly been addressed independently of one another. Studies have primarily examined nursing students’ levels of digital literacy and clinical decision-making skills, the factors influencing these skills, and related variables (e.g., emotional intelligence, critical thinking) (6, 16–21). However, it appears that studies examining the relationship between digital literacy and clinical decision-making skills among nursing students are limited. This knowledge gap necessitates a more detailed investigation of the potential contribution of digital literacy to students’ clinical decision-making processes. Establishing this relationship could contribute to the development of educational strategies that address digital literacy and clinical decision-making skills through a holistic approach within nursing education programs. Furthermore, the results are expected to contribute to the planning of educational programs that will support the professional competencies of future nurses during the digital transformation process. Therefore, this study aimed to examine the relationship between the digital literacy levels and clinical decision-making skills of nursing students at a public university.

## 2 Methods

### 2.1 Study population

This study employed a descriptive, cross-sectional, and correlational design. The target population consisted of 734 nursing students enrolled in the nursing department of a public university. The required minimum sample size was calculated as 253 using the sample size estimation formula for a finite population, assuming a 95% confidence level and a 0.05 margin of error. To account for potential nonresponse and missing data, participant selection was continued beyond the required minimum sample size. A convenience sampling method was used to recruit participants. All nursing students who met the eligibility criteria were invited to participate in the study. The inclusion criteria were being over 18 years of age and volunteering to participate in the study. Nursing students who did not wish to participate and those under 18 years of age were not included in the study. A total of 276 students consented to participate and completed the data collection forms. Of these, five participants were excluded from the analysis because their data collection forms were incomplete. Therefore, the final sample consisted of 271 nursing students, which exceeded the minimum required sample size of 253. The participant flow was as follows: the target population consisted of 734 nursing students; 276 students consented to participate and provided data; five participants were excluded due to incomplete data collection forms; and 271 participants were included in the final analysis. In reporting this study, the Strengthening the Reporting of Observational Studies in Epidemiology (STROBE) guidelines for cross-sectional studies were followed.

### 2.2 Survey instrument

The following tools were used to collect research data: The Information Form, the Digital Literacy Scale, and the Clinical Decision-Making in Nursing Scale.

#### 2.2.1 Information form

The form contains eight questions designed to determine the nursing students’ ages, genders, class levels, daily internet usage times, purposes of internet use, digital devices they own, their level of perceived responsibility for decision-making in clinical practice, and their sense of competence regarding clinical decision-making.

#### 2.2.2 Digital literacy scale

The original scale was developed by Ng in 2012 (22). Adapted into Turkish by Hamutoğlu et al. (23), the scale consists of 17 items and 4 subscales (attitudinal, technical, cognitive, and social). It is structured as a Likert-type scale (5-point rating). Each item is scored on a scale from 1 (Strongly Disagree) to 5 (Strongly Agree). The lowest possible score on the attitude subscale is 7, and the highest is 35. On the technical subscale, the lowest possible score is 6, and the highest is 30; on the cognitive subscale, the lowest is 2, and the highest is 10; and on the social subscale, the lowest is 2, and the highest is 10. A higher score indicates higher digital literacy. The scale’s overall Cronbach’s alpha coefficient was reported as 0.93 (23). In this study, it was determined to be 0.73. The Digital Literacy Scale demonstrated acceptable internal consistency in the present study, although this coefficient was lower than that reported in the Turkish adaptation study (23). This difference may be related to variations in sample characteristics and study context, as internal consistency estimates may vary across different populations.

#### 2.2.3 The clinical decision-making in nursing scale

The scale was developed by Jenkins in 1983 to help students describe their perceptions of clinical decision-making in their own words (24). A study on the scale’s reliability and validity in Turkish was conducted by Durmaz Edeer and Sarıkaya (25). The scale consists of 40 items and 4 subscales. Scores range from 40 to 200 on the total scale and from 10 to 50 on each subscale; there is no cutoff point. A high score on the scale indicates a high perception of clinical decision-making, while a low score indicates a low perception of clinical decision-making. The total Cronbach’s alpha internal consistency coefficient for the original version of the scale was α = 0.83 (24); in the Turkish adaptation study, it was 0.78 (25); and in this study, it was determined to be 0.91.

### 2.3 Data collection

The data for this study were collected by the researcher using face-to-face data collection methods between June 2 and June 19, 2026, from nursing students who met the inclusion criteria. Before the data collection process began, the purpose and scope of the study, the procedures to be followed, the principle of voluntary participation, and the fact that participants could withdraw from the study at any time without providing a reason were verbally explained to the participants. Written informed consent was obtained from the students who agreed to participate in the study. Data collection instruments were distributed to participants in printed form, and students completed the forms individually outside of class hours, in a quiet and appropriate environment, free from the researcher’s influence. The completed forms were submitted to the researcher in sealed envelopes. This standardized data collection approach was designed to protect participant privacy and minimize social desirability bias and researcher influence. It took each participant approximately 20 min to complete the data collection instruments. Throughout the entire study process, participants’ identifying information was not collected; data were collected anonymously and used solely for scientific research purposes.

### 2.4 Statistical analysis

IBM SPSS Statistics for Windows, Version 27.0 (IBM Corp., Armonk, NY, USA) was used to analyze the data obtained in the study. When evaluating the study data, quantitative variables were presented using mean, standard deviation, median, minimum, and maximum values, while qualitative variables were presented using descriptive methods such as frequency and percentage. The suitability of the data for a normal distribution was examined using the Shapiro-Wilks test, box plot graphs, and skewness and kurtosis values. The skewness and kurtosis values for the subdimensions and total scores of the Digital Literacy Scale and the Clinical Decision-Making Scale in Nursing fell within the range of −3 to +3, indicating no substantial deviation from normality. For comparisons between two quantitative groups showing a normal distribution, Student’s t-test was used; for comparisons involving three or more groups, one-way ANOVA was used, and the Bonferroni *post hoc* test was used to identify the source of significant differences. For comparisons between two groups involving variables that did not follow a normal distribution, the Mann–Whitney *U* test was used. Pearson correlation analysis was performed to assess relationships between variables, depending on their distribution. Multiple linear regression analysis was conducted to examine the effect of the digital literacy scale characteristics on the total score of the Clinical Decision-Making in Nursing Scale. Effect sizes were calculated and reported as Cohen’s d for comparisons between two groups and eta squared (η^2^) for one-way ANOVA. Results were evaluated at a 95% confidence interval and a significance level of *p* < 0.05.

## 3 Results

Of the 271 nursing students who participated in the study, 74.9% (*n* = 203) were female, and 25.1% (*n* = 68) were male. The students’ ages ranged from a minimum of 18 to a maximum of 26, with a mean of 20.11 ± 1.68. Of the students, 25.1% (*n* = 68) were first-year students, 23.2% (*n* = 63) were second-year students, 27.3% (*n* = 74) were third-year students, and 24.4% (*n* = 66) were fourth-year students. Students’ daily internet usage ranges from 2 to 18 h, with an average of 6.07 ± 2.10 h. Their purposes for using the internet include 90.0% (*n* = 244) for coursework/research, 98.5% (*n* = 267) for social media, 95.2% (*n* = 258) for messaging/communication, 83.0% (*n* = 225) for entertainment/gaming/watching movies, and 54.2% (*n* = 147) for searching for health information. Regarding the digital devices owned by the students, it was determined that 99.3% (*n* = 269) own a smartphone, 64.2% (*n* = 174) own a tablet, 53.9% (*n* = 146) own a laptop, and 54.2% (*n* = 19) own a smartwatch. 7.4% of the students (*n* = 20) reported a low level of responsibility for decision-making in clinical practice, 75.3% (*n* = 204) reported a moderate level, and 17.3% (*n* = 47) reported a high level. 34.7% of the students (*n* = 94) reported feeling competent in clinical decision-making, 13.3% (*n* = 36) reported feeling incompetent, and 52.0% (*n* = 141) reported feeling partially competent.

Nursing students’ scores on the “Attitude” subscale of the Digital Literacy Scale averaged 27.54 ± 5.11 points; on the “Technical” subscale, 22.86 ± 4.26 points; 7.59 ± 1.43 on the “Cognitive” subscale, and 7.41 ± 1.58 on the “Social” subscale. The average total score on the Digital Literacy Scale was 65.40 ± 10.62 (Table 1).

**Table 1:** Mean scores for the subscales and total scores of the Clinical Decision-Making in Nursing Scale and the Digital Literacy Scale among nursing students.

Scales	Mean ± SD	Min-Max	Skewness	Kurtosis
Attitude	27.54 ± 5.11	7–35	−1.204	2.069
Technical	22.86 ± 4.26	6–30	−0.841	2.062
Cognitive	7.59 ± 1.43	2–10	−0.801	2.033
Social	7.41 ± 1.58	2–10	−0.785	1.029
Total digital literacy scale	65.40 ± 10.62	17–85	−1.210	2.118
Search for alternatives or options	35.75 ± 4.64	25–50	0.274	−0.163
Canvassing of objectives and values	33.20 ± 3.13	25–44	0.277	0.304
Evaluation and reevaluation of consequences	35.47 ± 4.76	24–49	0.255	−0.544
Search for information and unbiased assimilation of new information	33.07 ± 3.71	24–43	0.322	−0.285
Total The Clinical Decision-Making in Nursing Scale	137.49 ± 14.04	102–173	0.325	−0.518

Min: Minimum. Max: Maximum. SD: Standard Deviation.

Nursing students scored an average of 35.75 ± 4.64 points on the “Search for Alternatives or Options” subscale of The Clinical Decision-Making in Nursing Scale; 33.20 ± 3.13 points on the “Canvassing of Objectives and Values” subscale; 35.47 ± 4.76 points on the “Evaluation and Reevaluation of Consequences” subscale, and 33.07 ± 3.71 points on the “Search for Information and Unbiased Assimilation of New Information” subscale. The mean total scores on the Clinical Decision-Making in Nursing Scale were 137.49 ± 14.04 (Table 1).

Nursing students’ Digital Literacy Scale scores showed significant differences in terms of gender, grade level, daily internet usage time, and purpose of internet use (all *p* < 0.05). No statistically significant relationship was found between the nursing students’ ages and the Digital Literacy Scale subscales or total scores (*p* > 0.05) (Table 2).

**Table 2:** Comparison of nursing students’ descriptive characteristics with their Digital Literacy Scale scores.

Characteristic/Variables		*n*	Digital literacy scale				
			Attitude	Technical	Cognitive	Social	Total
			Mean ± SD	Mean ± SD	Mean ± SD	Mean ± SD	Mean ± SD
Gender	Female	203	27.72 ± 5.15	22.48 ± 4.40	7.55 ± 1.47	7.43 ± 1.54	65.18 ± 10.99
	Male	68	27.00 ± 4.98	24.00 ± 3.57	7.69 ± 1.30	7.35 ± 1.71	66.04 ± 9.49
Test value/*p*			*t:1.005/^a^0.316*	t:–2.571/^a^0.011*	*t:–0.697/^a^0.486*	*t:0.340/^a^0.734*	*t:–0.579/^a^0.563*
Effect size / 95% CI			*d* = 0.141/−0.134–0.416	*d* = −0.360/−0.637 – −0.084	*d* = −0.098/−0.372–0.177	*d* = 0.048/−0.227–0.322	*d* = −0.081/−0.356–0.194
Age		*r*	0.066	0.013	0.009	−0.081	0.026
		*p*	0.281	0.832	0.881	0.185	0.670
95% CI			−0.054 – 0.184	−0.106 – 0.132	−0.110 – 0.128	−0.198 – 0.039	−0.093 – 0.145
Grade	1st Grade	68	26.06 ± 5.11	22.06 ± 3.60	7.40 ± 1.37	7.38 ± 1.53	62.90 ± 9.70
	2nd Grade	63	28.16 ± 4.28	23.65 ± 4.25	7.70 ± 1.39	7.81 ± 1.46	67.32 ± 9.69
	3rd Grade	74	27.31 ± 5.87	22.68 ± 5.00	7.49 ± 1.69	7.04 ± 1.76	64.51 ± 12.68
	4th Grade	66	28.73 ± 4.59	23.15 ± 3.89	7.79 ± 1.17	7.47 ± 1.48	67.14 ± 9.33
Test value/*p*			F:3.553/^b^0.015*	*F:1.691/^b^0.169*	*F:1.089/^b^0.354*	F:2.769/^b^0.042*	F:2.757/^b^0.043*
Effect size / 95% CI			η^2^ = 0.038/0.001–0.084	η^2^ = 0.019/0.000–0.053	η^2^ = 0.012/0.000–0.040	η^2^ = 0.030/0.000–0.072	η^2^ = 0.030/0.000–0.072
Daily internet usage time		r	0.151	0.110	0.090	0.124	0.147
		p	0.013*	0.070	0.140	0.042*	0.015*
95% CI			0.032–0.265	−0.009 – 0.226	−0.029 – 0.207	0.005–0.240	0.028–0.262
Purpose of internet use							
Coursework/ Research	Yes	244	27.65 ± 5.15	22.85 ± 4.33	7.60 ± 1.44	7.39 ± 1.58	65.49 ± 10.78
	No	27	26.56 ± 4.64	23.00 ± 3.58	7.48 ± 1.37	7.56 ± 1.67	64.59 ± 9.15
Test value/*p*			*t:1.055/^a^0.293*	*t:-0.175/^a^0.861*	*t:0.403/^a^0.687*	*t:-0.504/^a^0.615*	*t:0.415/^a^0.678*
Effect size / 95% CI			*d* = 0.214/−0.184–0.612	*d* = −0.035/−0.433–0.362	*d* = 0.082/−0.316–0.479	*d* = −0.102/−0.500–0.295	*d* = 0.084/−0.313–0.482
Social media	Yes	267	27.59 ± 4.99	22.90 ± 4.15	7.60 ± 1.41	7.42 ± 1.56	65.50 ± 10.30
	No	4	24.00 ± 10.98	20.50 ± 9.75	7.00 ± 2.58	7.00 ± 2.94	58.50 ± 26.16
Test value/*p*			*Z:-0.576/^c^0.565*	*Z:-0.233/^c^0.816*	*Z:-0.543/^c^0.587*	*Z:-0.137/^c^0.891*	*Z:-0.386/^c^0.699*
Effect size / 95% CI			*d* = −0.035/−0.154–0.085	*d* = −0.014/−0.133–0.105	*d* = −0.033/−0.152–0.087	*d* = −0.008/−0.127–0.111	*d* = −0.023/−0.142–0.096
Messaging/ Communication	Yes	258	27.54 ± 5.17	22.80 ± 4.30	7.57 ± 1.43	7.40 ± 1.60	65.30 ± 10.72
	No	13	27.54 ± 3.76	24.15 ± 3.13	7.92 ± 1.32	7.69 ± 1.32	67.31 ± 8.47
Test value/*p*			*t:0.000/^a^1.000*	*t:-1.121/^a^0.263*	*t:-0.871/^a^0.385*	*t:-0.659/^a^0.511*	*t:-0.664/^a^0.507*
Effect size / 95% CI			*d* = 0.000/−0.557–0.557	*d* = −0.319/−0.876–0.239	*d* = −0.248/−0.805–0.310	*d* = −0.187/−0.745–0.370	*d* = −0.189/−0.746–0.369
Entertainment/ Gaming/Watching movies	Yes	225	27.84 ± 4.89	23.07 ± 4.07	7.62 ± 1.38	7.49 ± 1.53	66.03 ± 9.91
	No	46	26.04 ± 5.89	21.85 ± 4.99	7.41 ± 1.65	7.00 ± 1.80	62.30 ± 13.27
Test value/*p*			t:2.195/^a^0.029*	*t:1.784/^a^0.076*	*t:0.906/^a^0.366*	*t:1.934/^a^0.054*	t:2.184/^a^0.030*
Effect size / 95% CI			*d* = 0.355/0.037–0.674	*d* = 0.289/−0.029–0.607	*d* = 0.147/−0.171–0.464	*d* = 0.313/−0.005–0.631	*d* = 0.353/0.035–0.672
Searching for health information	Yes	147	28.18 ± 4.92	23.21 ± 4.07	7.70 ± 1.43	7.54 ± 1.59	66.63 ± 10.31
	No	124	26.77 ± 5.24	22.45 ± 4.45	7.45 ± 1.42	7.26 ± 1.57	63.94 ± 10.83
Test value/p			t:2.281/^a^0.023*	*t:1.466/^a^0.144*	*t:1.434/^a^0.153*	*t:1.449/^a^0.148*	t:2.096/^a^0.037*
Effect size / 95% CI			*d* = 0.278/0.038–0.518	*d* = 0.179/−0.061–0.418	*d* = 0.175/−0.065–0.414	*d* = 0.177/−0.063–0.416	*d* = 0.256/0.016–0.496

*r* = Pearson’s Correlation Test. *p < 0.05. *d* = Cohen’s *d*. η^2^ = Eta squared. ^a^Student’s *t*-test^. b^One-Way ANOVA Test and Bonferroni Test. ^c^Mann-Whitney *U* Test. CI = Confidence interval.

Bold values indicate statistically significant differences (*p* < 0.05).

Male students’ scores on the “Technical” subscale of the Digital Literacy Scale were found to be statistically significantly higher than those of female students (*p* = 0.011, *Cohen’s d* = −0.360, *95% CI* -0.637 to 0.084). Bonferroni post-hoc analysis showed that fourth-year nursing students’ scores on the “Attitude” subscale of the Digital Literacy Scale was higher than those of first-year students (*p* = 0.014, *eta squared* = −0.038, *95% CI* 0.001 to −0.084), second-year students’ “Social” subscale scores were higher than those of third-year students (*p* = 0.027, *eta squared* = 0.030, *95% CI* 0.000 to −0.072), and second-year students’ total scale scores were higher than those of first-year students (*p =* 0.048). A very weak positive correlation was found between daily internet usage time and the “Attitude,” “Social” subscale, and total scale scores (*p* < 0.05). In addition, higher “Attitude” and total scale scores were observed among students who used the internet for entertainment/gaming/watching movies and searching for health information (*p* < 0.05). In contrast, there were no significant differences in scale scores among students who used the internet for coursework/research, social media, and messaging/communication (Table 2).

Nursing students’ scores on the Clinical Decision-Making in Nursing Scale showed significant differences in terms of gender, daily internet usage time, and purpose of internet use (all *p* < 0.05; *p* < 0.01). No statistically significant relationship was found between the nursing students’ ages and the subscales or total scores of the Clinical Decision-Making in Nursing Scale (*p* > 0.05). Furthermore, no statistically significant difference was found between the subscales and total scores of the Clinical Decision-Making in Nursing Scale based on the nursing students’ class levels (*p* > 0.05) (Table 3).

**Table 3:** Comparison of nursing students’ descriptive characteristics with the Clinical Decision-Making in Nursing Scale scores.

Characteristic/Variables		*n*	The clinical decision-making in nursing scale				
			Search for alternatives or options	Canvassing of objectives and values	Evaluation and reevaluation of consequences	Search for information and unbiased assimilation of new information	Total
			Mean ± SD	Mean ± SD	Mean ± SD	Mean ± SD	Mean ± SD
Gender	Female	203	36.12 ± 4.82	33.66 ± 3.16	36.03 ± 4.79	33.55 ± 3.77	139.36 ± 14.42
	Male	68	34.63 ± 3.89	31.82 ± 2.58	33.79 ± 4.29	31.66 ± 3.14	131.91 ± 11.17
Test value/*p*			t:2.309/^a^0.022*	t:4.328/^a^0.001**	t:3.418/^a^0.001**	t:3.713/^a^0.001**	t:3.884/^a^0.001**
Effect size / 95% CI			*d* = 0.324/0.048–0.599	d = 0.606/0.327–0.886	d = 0.479/0.201–0.756	d = 0.520/0.242–0.798	d = 0.544/0.266–0.823
Age		*r*	0.097	0.099	0.077	0.067	0.098
		*p*	0.112	0.105	0.206	0.268	0.108
95% CI			−0.022 – 0.214	−0.020 – 0.216	−0.043 – 0.194	−0.053 – 0.185	−0.021 – 0.215
Grade	1^st^ Grade	68	34.54 ± 4.29	32.51 ± 2.52	34.12 ± 4.13	32.71 ± 3.08	133.88 ± 11.44
	2^nd^ Grade	63	36.02 ± 4.29	33.03 ± 3.01	36.08 ± 4.65	32.68 ± 3.27	137.81 ± 12.76
	3^rd^ Grade	74	36.01 ± 4.35	33.53 ± 2.95	35.81 ± 4.82	33.23 ± 3.85	138.58 ± 14.06
	4^th^ Grade	66	36.44 ± 5.44	33.7 ± 3.84	35.89 ± 5.21	33.65 ± 4.46	139.68 ± 16.92
Test value/p			*F:2.189/^b^0.090*	*F:1.997/^b^0.115*	*F:2.517/^b^0.059*	*F:1.035/^b^0.378*	*F:2.223/^b^0.086*
Effect size / 95% CI			η^2^ = 0.024/0.000–0.062	η^2^ = 0.022/0.000–0.058	η^2^ = 0.028/0.000–0.068	η^2^ = 0.011/0.000–0.039	η^2^ = 0.024/0.000–0.062
Daily internet usage time		*r*	−0.105	0.040	−0.119	−0.170	−0.111
		*p*	0.085	0.511	0.050	0.005**	0.068
95% CI			−0.221 – 0.014	−0.080 – 0.158	−0.235 – 0.000	−0.283 – −0.052	−0.227 – 0.008
Purpose of internet use							
Coursework/ Research	Yes	244	35.79 ± 4.57	33.28 ± 3.11	35.61 ± 4.71	33.14 ± 3.75	137.82 ± 13.92
	No	27	35.41 ± 5.34	32.44 ± 3.27	34.15 ± 5.05	32.52 ± 3.37	134.52 ± 15.04
Test value/p			*t:0.402/^a^0.688*	*t:1.324/^a^0.187*	*t:1.523/^a^0.129*	*t:0.820/^a^0.413*	*t:1.160/^a^0.247*
Effect size / 95% CI			*d* = 0.082/−0.316–0.479	*d* = 0.269/−0.130–0.667	*d* = 0.309/−0.089–0.707	*d* = 0.166/−0.231–0.564	*d* = 0.235/−0.163–0.633
Social media	Yes	267	35.75 ± 4.61	33.21 ± 3.12	35.48 ± 4.74	33.09 ± 3.67	137.54 ± 13.91
	No	4	35.5 ± 7.42	32.25 ± 4.19	34.5 ± 6.81	32.25 ± 6.55	134.5 ± 23.91
Test value/p			*Z:-0.493/^c^0.622*	*Z:-0.740/^c^0.459*	*Z:-0.535/^c^0.593*	*Z:-1.103/^c^0.270*	*Z:-0.961/^c^0.336*
Effect size / 95% CI			−0.149 – 0.090	−0.163 – 0.075	−0.151 – 0.087	−0.185 – 0.053	−0.176 – 0.061
Messaging/ Communication	Yes	258	35.69 ± 4.58	33.20 ± 3.12	35.43 ± 4.72	33.07 ± 3.71	137.39 ± 13.86
	No	13	36.85 ± 5.90	33.23 ± 3.35	36.31 ± 5.54	33.08 ± 3.90	139.46 ± 17.69
Test value/p			*t:-0.872/^a^0.384*	*t:-0.037/^a^0.970*	*t:-0.651/^a^0.516*	*t:-0.003/^a^0.998*	*t:-0.518/^a^0.605*
Effect size / 95% CI			*d* = −0.248/−0.805–0.310	*d* = −0.011/−0.568–0.547	*d* = −0.185/−0.742–0.372	*d* = −0.001/−0.558–0.556	*d* = −0.147/−0.705–0.410
Entertainment/ Gaming/Watching movies	Yes	225	35.97 ± 4.46	33.36 ± 3.03	35.45 ± 4.72	33.08 ± 3.61	137.87 ± 13.54
	No	46	34.65 ± 5.36	32.39 ± 3.50	35.57 ± 5.00	33.04 ± 4.21	135.65 ± 16.30
Test value/p			*t:1.765/^a^0.079*	*t:1.933/^a^0.054*	*t:-0.151/^a^0.880*	*t:0.061/^a^0.952*	*t:0.975/^a^0.331*
Effect size / 95% CI			*d* = 0.286/−0.032–0.604	*d* = 0.313/−0.005–0.631	*d* = −0.024/−0.342–0.293	*d* = 0.010/−0.307–0.327	*d* = 0.158/−0.160–0.475
Searching for health information	Yes	147	36.30 ± 4.55	33.72 ± 3.11	35.99 ± 4.69	33.39 ± 3.71	139.40 ± 13.92
	No	124	35.10 ± 4.69	32.58 ± 3.05	34.85 ± 4.78	32.69 ± 3.68	135.23 ± 13.90
Test value/p			t:2.137/^a^0.033*	t:3.037/^a^0.003**	*t:1.960/^a^0.051*	*t:1.555/^a^0.121*	t:2.462/^a^0.014*
Effect size / 95% CI			*d* = 0.261/0.021–0.501	*d* = 0.370/0.129–0.611	*d* = 0.239/−0.001–0.479	*d* = 0.190/−0.050–0.429	*d* = 0.300/0.060–0.541

*r* = Pearson’s Correlation Test. SD: Standard Deviation. *d* = Cohen’s *d*. η^2^ = Eta squared. ^a^Student’s *t*-test^. b^One-Way ANOVA Test and Bonferroni Test. ^c^Mann-Whitney *U* Test. *p < 0.05, **p < 0.01. CI = Confidence interval.

Bold values indicate statistically significant differences (*p* < 0.05).

Female students’ total scores and scores on all subscales of the Clinical Decision-Making in Nursing Scale were found to be statistically significantly higher than those of male students (*p* = 0.022; *p* = 0.001; *p* = 0.001; *p* = 0.001; *p* = 0.001). A very weak negative correlation was found between daily internet usage duration and scores on the “Search for Information and Unbiased Assimilation of New Information” subscale (*p* = 0.005). In addition, students who used the internet to search for health information were found to have higher scores on “Search for Alternatives or Options,” “Canvassing of Objectives and Values,” and the total scale score (*p =* 0.033; *p* = 0.003; *p* = 0.014). In contrast, scale scores did not show significant differences among students who used the internet for coursework/research, social media, messaging/communication, and entertainment/gaming/watching movies (Table 3).

The study found a weak to moderate positive and statistically significant relationship between nursing students’ Digital Literacy Scale “Attitude,” “Technical,” and “Cognitive” subscale scores and total scores, and the mean total and subscale scores of The Clinical Decision-Making in Nursing Scale (*r* = 0.129–0.335, *p* ≤ 0.033) (Table 4).

**Table 4:** Relationship between nursing students’ Digital Literacy Scale scores and the Clinical Decision-Making in Nursing Scale scores.

The clinical decision-making in nursing scale		Digital literacy scale				
Subscales	Statistical Values	Attitude	Technical	Cognitive	Social	Total
Search for alternatives or options	*r*	0.305	0.261	0.234	0.101	0.298
	*p*	0.001**	0.001**	0.001**	0.098	0.001**
95% CI		0.193–0.409	0.146–0.369	0.118–0.344	−0.018 – 0.218	0.185–0.403
Canvassing of objectives and values	*r*	0.335	0.246	0.255	0.136	0.314
	*p*	0.001**	0.001**	0.001**	0.025*	0.001**
95% CI		0.225–0.437	0.131–0.355	0.140–0.363	0.017–0.251	0.202–0.418
Evaluation and reevaluation of consequences	*r*	0.262	0.160	0.184	0.004	0.215
	*p*	0.001**	0.009**	0.002**	0.949	0.001**
95% CI		0.147–0.370	0.042–0.274	0.066–0.297	−0.115 – 0.123	0.098–0.326
Search for information and unbiased assimilation of new information	*r*	0.261	0.129	0.137	0.035	0.201
	*p*	0.001**	0.033*	0.024*	0.564	0.001**
95% CI		0.146–0.369	0.010–0.244	0.018–0.252	−0.085 – 0.154	0.084–0.313
Total	*r*	0.333	0.230	0.233	0.074	0.295
	*p*	0.001**	0.001**	0.001**	0.223	0.001**
95% CI		0.223–0.435	0.114–0.340	0.117–0.343	−0.046 – 0.191	0.182–0.400

**p < 0.01, *p < 0.05 CI = Confidence interval. r: Pearson’s correlation analysis.

In the study, multiple linear regression analysis was conducted to examine the effects of the subscales of the Digital Literacy Scale on the total score of the Clinical Decision-Making in Nursing Scale. The results regarding the resulting regression model are presented in Table 5.

**Table 5:** Effects of the subscales of the Digital Literacy Scale on the total score of the Clinical Decision-Making in Nursing Scale.

Scale	Independent variables	Unstandardized *ß*	Std. error	*ß*	*t*	*p*	%95 GA	Tolerance	VIF
Digital literacy scale total score	(Constant)	113.151	5.133		22.042	0.001**	103.044–123.259		
	Attitude	0.946	0.220	0.344	4.294	0.001**	0.512–1.380	0.512	1.954
	Technical	−0.101	0.334	−0.031	−0.302	0.763	−0.758-0.557	0.320	3.121
	Cognitive	1.172	0.882	0.119	1.330	0.185	−0.563-2.908	0.409	2.443
	Social	−1.120	0.610	−0.126	−1.837	0.067	−2.321-0.081	0.694	1.441

Dependent variable: Clinical Decision-Making in Nursing Scale total score **p < 0.01. *F*(4.266) = 9.584; *p* = 0.001; *R*^2^ = 0.126; Adjusted *R*^2^ = 0.113.

The multiple linear regression model developed to predict the total score on The Clinical Decision-Making in Nursing Scale was found to be statistically significant (*F(4.266)* = 9.584; *p* = 0.001). The model explained 12.6% of the variance in the total score on the Clinical Decision-Making in Nursing Scale (*R^2^* = 0.126; *adjusted R^2^* = 0.113). Among the digital literacy scale subdimensions, only the Attitude subdimension was a significant predictor of the total score on The Clinical Decision-Making in Nursing Scale (*B* = 0.946, *SE* = 0.220, *β*: 0.344; *95% CI* [0.512,1.380], *t* = 4.294, *p* = 0.001). Higher Attitude scores were associated with higher clinical decision-making scores. The Technical, Cognitive, and Social subdimensions were not significant predictors (*p* > 0.05). Multicollinearity was not considered problematic, as tolerance values ranged from 0.320 to 0.694 and VIF values ranged from 1.441 to 3.121 (Table 5 and Figure 1).

**Figure 1:**
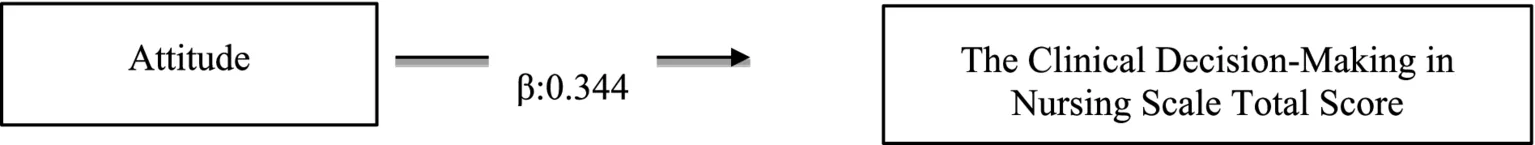
Results model showing the effects of the Digital Literacy Scale Attitude score on the total score of the Clinical Decision-Making in Nursing Scale.

It was determined that the scores for the Technical, Cognitive, and Social subscales were not significant predictors of the total score on the Clinical Decision-Making in Nursing Scale (all *p* > 0.05) (Table 5).

## 4 Discussion

In this study, which was conducted to examine the relationship between nursing students’ levels of digital literacy and their clinical decision-making skills, it was found that nearly all nursing students own a digital device and use the internet for an average of 6 h a day. Similarly, a study conducted with nursing students in Egypt found that students used the internet for more than 4 h a day (26), while studies conducted among recent university graduates (27) and medical students (28) reported that the majority of participants used the internet for 5 h or less per day. Additionally, Zafra-Agea et al. (29) reported that nursing students used the internet for more than 20 to 40 h per week. Based on these results, it can be said that digital technologies have become an integral part of nursing students’ daily lives.

This study found that nursing students most frequently used the internet for coursework/research, social media, messaging/communication, entertainment/gaming/watching movies, and searching for health information. Similarly, Lokmic-Tomkins et al. (30) noted that nursing students primarily use the internet for information search, online social networking, and watching videos, while Erdat et al. (20) reported that they use it for social media, education, research, news, and games. When these results are evaluated together, they indicate that nursing students primarily use the internet for coursework, research, and searching for health information, demonstrating that digital environments are embraced not only for communication and entertainment but also as a significant source of information in their professional learning processes. This situation highlights the importance of developing digital literacy skills so that future nurses can effectively utilize information obtained from digital environments.

This study found that nursing students’ digital literacy levels were above average. This result is consistent with the results reported by Erdat et al. (20). In contrast, Brown et al. (4) found that while nursing students’ daily digital literacy levels were high, their ability to apply this skill in clinical settings was low, whereas Martzoukou et al. (6) determined that their digital literacy levels were low. It is thought that the differences in the results obtained from these studies may stem from variations in sample characteristics, the level of digital technology use in educational programs, and differences in students’ access to digital learning environments. In this study, the fact that nearly all students own a digital device and use the internet for an average of 6 h a day indicates that they interact frequently with digital technologies. The literature indicates that greater interaction with digital technologies may contribute to the development of digital skills (31, 32). In this context, the fact that the students’ digital literacy levels in our study were above the intermediate level may be related to their access to and experience with digital technologies. Nursing students need to possess an adequate level of digital literacy to access and evaluate information in digital health environments and to use it appropriately in professional practice.

In this study, male nursing students scored higher on the technical subscale of digital literacy than female students. This result is consistent with the results of Aydın Kahraman’s study (33). Nguyen and Habók (34) also found that male students had higher technical skills than female students. In contrast, Zhou et al. (35) found no significant difference in digital literacy based on gender among university students. In a literature review conducted by Peng and Yu (36), it was reported that gender is a factor influencing digital literacy, though different studies have yielded varying results. These results can be explained by the increasing access to and opportunities for using digital technologies, as well as the growing similarity in these opportunities. Therefore, it is thought that these differing results regarding the relationship between gender and digital literacy may be influenced by students’ socioeconomic status, individual interests, education, and environmental factors.

In this study, a positive relationship was found between nursing students’ daily internet usage time and their levels of digital literacy. Additionally, it was determined that nursing students who used the internet for entertainment, gaming, watching movies, and searching for health information had higher levels of digital literacy. Regardless of whether students use digital technologies for educational or recreational purposes, their increased interaction with the internet and digital devices helps them adapt more easily to the online environment (37). This suggests that increased interaction with digital technologies may contribute to the development of digital literacy skills. In particular, it can be argued that using the internet to search for health information supports skills such as accessing information, critically evaluating the information obtained, and distinguishing reliable information sources. Indeed, in a study by Zafra-Agea et al. (29), it was noted that the majority of nursing students search for health-related information online and that the vast majority know how to use this information. However, it should be noted that it is the purpose and nature of internet use—rather than merely its frequency—that is associated with the level of digital literacy.

In this study, it was determined that there were significant differences in the digital literacy levels of nursing students based on their year of study. Similarly, the study by Bahri et al. (38) also found a significant relationship between students’ year of study and their digital literacy levels. The fact that second- and fourth-year nursing students’ digital literacy scores were found to be higher than those of first-year students in our study suggests that increased exposure to and experience with digital technologies during the educational process may contribute to the development of digital literacy. This result is consistent with the study conducted by Aydınlar et al. (39) with students in the health sciences, which reported that fourth-year students’ digital literacy levels were higher than those of first-year students. However, the fact that second-year students’ scores on the social subscale and the total scale were higher than those of third-year students suggests that digital literacy cannot be explained solely by grade level and may not show a linear increase as students’ progress through their studies. Therefore, it can be argued that digital literacy should be evaluated in conjunction with the experiences students gain throughout their education, as well as their purposes for using digital technologies, individual characteristics, experiences, and digital opportunities in their learning environments.

It was determined that nursing students’ perceptions of clinical decision-making were at a moderate level. Similarly, studies by Amin et al. (18) and Arkan et al. (19) found that nursing students’ clinical decision-making skills were at a moderate level. In contrast, studies by Jawabreh (21) and Alharbi et al. (40) found that nursing students’ clinical decision-making skills were high. The differences among these studies may stem from variations in students’ clinical practice experiences and the educational methods used to support clinical decision-making. Since clinical decision-making is one of the fundamental skills of effective and safe nursing care, it is of great importance for students to develop this skill during their education (16). In this regard, the result that perceptions of clinical decision-making were at a moderate level in this study suggests a need to strengthen evidence-based learning activities and practices aimed at developing students’ clinical decision-making skills in undergraduate nursing education.

The study found a significant relationship between nursing students’ gender and their perceptions of clinical decision-making, and those female students’ perceptions of clinical decision-making were higher than those of male students. This result is consistent with some studies reporting that gender may influence clinical decision-making processes in health care delivery and nursing education. Indeed, Alharbi et al. (40) also reported that female students had higher clinical decision-making scores. In contrast, Arkan et al. (19) reported that male nursing students had higher clinical decision-making skill scores than female students, while Alzamel et al. (17) reported that male nurses had higher clinical decision-making skill scores than female nurses. These differences among the studies may stem from variations in sample characteristics, cultural context, and clinical training environments.

In this study, a very weak negative correlation was found between nursing students’ daily internet usage time and the “searching for information and objectively assimilating new information” subscale of clinical decision-making skills. The weakness of this correlation suggests that daily internet usage time is only weakly associated with clinical decision-making skills. Today, students use the internet for various purposes in both their educational and social lives. This study and previous research have shown that students use the internet not only for educational purposes but also for social media, communication, and entertainment (30, 41). This diversity of usage purposes may cause total internet usage time to reflect the form of usage rather than learning outcomes. Consequently, it suggests that the purpose and manner of internet use—rather than the duration of internet use—may be more decisive in terms of clinical decision-making skills.

The study found that nursing students who used the internet to search for health information had higher perceptions of clinical decision-making. Clinical decision-making is a multidimensional skill that involves the systematic evaluation of patient data, the critical analysis of available evidence, and the integration of the information obtained into patient-centered decision-making processes (42). In their study, Zafra-Agea et al. (29) reported that the majority of nursing students rely on information found online when making health-related decisions. In this regard, it is thought that the higher clinical decision-making perceptions among students who use the internet to search for health information may be related to their being more active in health-related information-seeking processes and interacting with various information sources. Conversely, since using the internet for coursework/research, social media, messaging/communication, or entertainment was not found to be associated with perceptions of clinical decision-making, it is thought that the purpose and nature of internet use—rather than the quantity of use—may be more closely related to perceptions of clinical decision-making.

In this study, weak to moderate positive and statistically significant correlations were found between nursing students’ levels of digital literacy and their clinical decision-making skills. Multiple linear regression analysis revealed that only the “Attitude” subscale of digital literacy was a significant predictor of perceived clinical decision-making skills, and that the model explained 12.6% of the variance in clinical decision-making scores. This result suggests that a positive attitude toward digital technologies may be associated with nursing students being more open to utilizing reliable digital information sources and applying this information in clinical decision-making processes.

Since digital health technologies are an integral part of healthcare services, nurses must possess not only technical digital skills but also the ability to access, evaluate, interpret, and effectively use digital information in clinical practice (43). For this reason, digital literacy is considered a multidimensional competency that goes beyond technical proficiency to include positive attitudes and motivation toward using digital technologies (6). Indeed, it has been reported that students’ positive attitudes toward digital technologies support their engagement in learning processes, innovative behaviors, and technology use (44). Furthermore, it has been demonstrated that digital literacy among nursing students is positively associated with clinical competence and performance (45, 46). In this context, the result in our study that only the attitude subscale of digital literacy predicts clinical decision-making skills can be explained by the fact that a positive attitude toward digital technologies supports students’ tendencies to use reliable digital information sources, actively participate in learning processes, and integrate this information into clinical practice.

However, the fact that the technical, cognitive, and social subscales of digital literacy did not significantly predict clinical decision-making ability in our study indicates that clinical decision-making ability is a multidimensional construct that cannot be explained by digital literacy alone. Clinical decision-making is a cognitive process that requires the systematic collection and analysis of patient data, the critical evaluation of evidence, the comparison of alternatives, and the determination of the most appropriate care decision tailored to the individual (47, 48). This process is associated with numerous individual and environmental factors, such as theoretical knowledge, clinical experience, critical thinking, clinical reasoning, problem-solving skills, self-confidence, and the educational environment (16, 42). The results of this study indicate that digital literacy—particularly the subscale of positive attitudes toward digital technologies—is one of the key components supporting clinical decision-making; however, digital literacy alone is not sufficient for developing clinical decision-making skills. Initiatives aimed at developing digital literacy in nursing education, when implemented alongside educational strategies that support critical thinking, clinical reasoning, and evidence-based practice, may contribute more significantly to the development of students’ clinical decision-making skills.

## 5 Conclusion

This study demonstrated that nursing students had above-moderate levels of digital literacy and moderate levels of perceived clinical decision-making ability. Students’ purposes for using the internet, particularly seeking health information, were associated with higher levels of digital literacy and perceptions of clinical decision-making. Furthermore, digital literacy was positively associated with clinical decision-making, and the attitude subscale of digital literacy emerged as a significant predictor of clinical decision-making ability. These results suggest that developing positive attitudes toward digital technologies may play an important role in preparing nursing students to effectively engage with digital health resources and support clinical decision-making processes.

Given the increasing integration of digital technologies into healthcare, nursing education should consider incorporating strategies that enhance not only students’ technical digital skills but also their ability and willingness to use digital resources effectively in professional contexts. Strengthening digital literacy within undergraduate nursing curricula may contribute to preparing future nurses for evidence-informed and digitally supported healthcare environments.

## 6 Limitations

This study has several limitations. First, because of its cross-sectional design, causal relationships between digital literacy and clinical decision-making cannot be established. Second, the study was conducted at a single public university; therefore, the results may not be generalizable to nursing students from other institutions or different educational and cultural settings. Third, data were collected using self-reported questionnaires, which may be subject to recall and social desirability bias despite efforts to ensure anonymity and privacy during data collection. Finally, although digital literacy was positively associated with clinical decision-making, the regression model explained only 12.6% of the variance, suggesting that additional individual, educational, and environmental factors not examined in this study may also influence clinical decision-making. Future multicenter and longitudinal studies are recommended to clarify these relationships further.

## Data Availability

The raw data supporting the conclusions of this article will be made available by the authors, without undue reservation.

## References

[1] Borges do Nascimento IJ Abdulazeem HM Vasanthan LT Martinez EZ Zucoloto ML Østengaard L . The global effect of digital health technologies on health workers’ competencies and health workplace: an umbrella review of systematic reviews and lexical-based and sentence-based meta-analysis. Lancet Digit Health. (2023) 5:e534–44. doi: 10.1016/S2589-7500(23)00092-4, 37507197 PMC10397356

[2] Kraus S Schiavone F Pluzhnikova A Invernizzi AC. Digital transformation in healthcare: analyzing the current state-of-research. J Bus Res. (2021) 123:557–67. doi: 10.1016/j.jbusres.2020.10.030

[3] Govender I. Digital literacy and STEM skills-what is the connection? A systematic review. Tech Know Learn. (2025) 31:1821–42. doi: 10.1007/s10758-025-09879-x

[4] Brown J Morgan A Mason J Pope N Bosco AM. Students nurses’ digital literacy levels. Comput Inform Nurs. (2020) 38:451–8. doi: 10.1097/CIN.0000000000000615, 33955370

[5] Mohammadnejad F Freeman S Klassen-Ross T Hemingway D Banner D. Impacts of technology use on the workload of registered nurses: a scoping review. J Rehabil Assist Technol Eng. (2023) 10:1–11. doi: 10.1177/20556683231180189, 37342268 PMC10278405

[6] Martzoukou K Luders ES Mair J Kostagiolas P Johnson N Work F . A cross-sectional study of discipline-based self-perceived digital literacy competencies of nursing students. J Adv Nurs. (2024) 80:656–72. doi: 10.1111/jan.15801, 37489586

[7] Amin SM El-fattah Mahgoub SA Tawfik AF Khalil DE El-Sayed AAI Atta MHR . Nursing education in the digital era: the role of digital competence in enhancing academic motivation and lifelong learning among nursing students. BMC Nurs. (2025a) 24:571. doi: 10.1186/s12912-025-03199-2, 40399954 PMC12093728

[8] Dermody G Wadsworth D El Haddad M Prichard R Benson A Benson T . Bridging the digital divide: a multi-method evaluation of nursing readiness for digital health technology. J Adv Nurs. (2026) 82:3752–66. doi: 10.1111/jan.70105, 40762402 PMC12994680

[9] Johansen ML O’Brien JL. Decision making in nursing practice: a concept analysis. Nurs Forum. (2016) 51:40–8. doi: 10.1111/nuf.12119, 25639525

[10] Banning M. A review of clinical decision making: models and current research. J Clin Nurs. (2008) 17:187–95. doi: 10.1111/j.1365-2702.2006.01791.x, 17331095

[11] Chen S-L Hsu H-Y Chang C-F Lin EC-L. An exploration of the correlates of nurse practitioners’ clinical decision-making abilities. J Clin Nurs. (2016) 25:1016–24. doi: 10.1111/jocn.13136, 26879045

[12] Jans C Bogossian F Andersen P Levett-Jones T. Examining the impact of virtual reality on clinical decision making-an integrative review. Nurse Educ Today. (2023) 125:105767. doi: 10.1016/j.nedt.2023.105767, 36906980

[13] Farčić N Barać I Lovrić R Pačarić S Gvozdanović Z Ilakovac V. The influence of self-concept on clinical decision-making in nurses and nursing students: a cross-sectional study. Int J Environ Res Public Health. (2020) 17:3059. doi: 10.3390/ijerph17093059, 32354029 PMC7246852

[14] Ghodsi Astan P Goli R Hemmati Maslakpak M Rasouli J Alilu L. The effect of evidence-based nursing education on nurses’ clinical decision making: a randomized controlled trial. Health Sci Rep. (2022) 5:e837. doi: 10.1002/hsr2.837, 36189417 PMC9488901

[15] Hants L Bail K Paterson C. Clinical decision-making and the nursing process in digital health systems: an integrated systematic. J Clin Nurs. (2023) 32:7010–35. doi: 10.1111/jocn.16823, 37485751

[16] İlaslan E Adıbelli D Teskereci G Üzen Cura Ş. Development of nursing students’ critical thinking and clinical decision-making skills. Teach Learn Nurs. (2023) 18:152–9. doi: 10.1016/j.teln.2022.07.004

[17] Alzamel LG Jallad ST Alsaqer K Alghabbashi MT. The mediating role of job rotation between clinical leadership skills and clinical decision-making among Jordanian nurses. J Nurs Manag. (2026) 2026:e3436144–8. doi: 10.1155/jonm/3436144, 42363792 PMC13309901

[18] Amin SM El-Sayed AAI Alsenany SA Atta MHR Morsy OMI Asal MGR. How clinical reasoning and decision-making competences influence the provision of empathic care among nursing students. Teach Learn Nurs. (2025b) 20:e550–9. doi: 10.1016/j.teln.2025.01.005

[19] Arkan B Yılmaz D Gökdere Çinar H Uzun R. Clinical decision-making levels of nursing students and affecting factors. Cyprus J Med Sci. (2022) 7:738–44. doi: 10.4274/cjms.2021.2857

[20] Erdat Y Sezer Ceren RE Ozdemir L Uslu-Sahan F Bilgin A. Influence of technical, cognitive and socio-emotional factors on digital literacy in nursing students assessed using structural equation modeling. Nurse Educ Today. (2023) 130:105937. doi: 10.1016/j.nedt.2023.105937, 37639879

[21] Jawabreh N. The relationship between the emotional intelligence and clinical decision making among nursing students. SAGE Open Nurs. (2024) 10:1–8. doi: 10.1177/23779608241272459, 39119200 PMC11307361

[22] Ng W. Can we teach digital natives digital literacy? Comput Educ. (2012) 59:1065–78. doi: 10.1016/j.compedu.2012.04.016

[23] Hamutoğlu NB Canan Güngören Ö Kaya Uyanık G Gür Erdoğan D. Adapting digital literacy scale into Turkish. Ege Egıtım Derg. (2017) 18:408–29. doi: 10.12984/egeefd.295306

[24] Jenkins HM. "Clinical decision making in nursing scale". In: Waltz CF Jenkins LS, editors. Measurement of Nursing Outcomes Volume: 1 Measuring Nursing Performance in Practice, Education and Research. USA: Springer Publishing Company (2001). p. 33–7.

[25] Durmaz Edeer A Dicle A. Reliability and validity of the Turkish version of clinical decision making in nursing scale (CDMNS) for undergraduate students of nursing. Int J Psychol Educ Stud. (2015) 2:1–9. doi: 10.17220/ijpes.2015.03.001

[26] Mohamed SAA El-Fattah MAA-HA Ragab OHG. The effect of digital citizenship and cyberloafing on under graduate nursing students academic performance. Egypt Nurs J. (2024) 20:414–24. doi: 10.4103/enj.enj_28_23

[27] Mamun MA Rafi MA Al Mamun AHMS Hasan MZ Akter K Hsan K . Prevalence and psychiatric risk factors of excessive internet use among northern Bangladeshi job-seeking graduate students: a pilot study. Int J Ment Health Addict. (2021) 19:908–18. doi: 10.1007/s11469-019-00066-5

[28] Sadeghi N Rezaeian S Janatolmakan M Mohammadi MM Heidarian P Khatony A. The relationship between sleep quality and nomophobia among medical students. BMC Med Educ. (2025) 25:1246. doi: 10.1186/s12909-025-07847-8, 40898217 PMC12403425

[29] Zafra-Agea JA Calvillo-Nuñez N Gil-Jiménez Hellín-Pijuan I. Perception of internet use in health decision-making among nursing students. Eur J Investig Health Psychol Educ. (2023) 13:810–9. doi: 10.3390/ejihpe13050061 PMC10217240 37232699

[30] Lokmic-Tomkins Z Choo D Foley P Dix S Wong P Brand G. Pre-registration nursing students’ perceptions of their baseline digital literacy and what it means for education: a prospective COHORT survey study. Nurse Educ Today. (2022) 111:105308. doi: 10.1016/j.nedt.2022.105308, 35240398

[31] Anderson TD Ogruk-Maz G Bell TJ III. Enhancing digital literacy in higher education: a comprehensive analysis of digital skill development among college students. J High Educ Theory Pract. (2025) 25:1–22. doi: 10.33423/jhetp.v25i3.7631

[32] Vodă AI Cautisanu C Grădinaru C Tănăsescu C de Moraes GHSM. Exploring digital literacy skills in social sciences and humanities students. Sustainability. (2022) 14:2483. doi: 10.3390/su14052483

[33] Aydın Kahraman H. An examination of the relationship between nursing students’ self-efficacy perceptions toward online technologies and their digital literacy levels. Gümüşhane Univ J Health Sci. (2026) 15:257–69. doi: 10.37989/gumussagbil.1731641

[34] Nguyen LAT Habók A. Digital literacy of EFL students: an empirical study in Vietnamese universities. Libri. (2022) 72:53–66. doi: 10.1515/libri-2020-0165

[35] Zhou X Sun K Zhu K Feng L Sun Q Zhong D. The impact of digital literacy on university students’ innovation capability: evidence from Ningbo, China. Front Psychol. (2025) 16:1548817. doi: 10.3389/fpsyg.2025.1548817, 40697730 PMC12282524

[36] Peng D Yu Z. A literature review of digital literacy over two decades. Educ Res Int. (2022) 2533413:1–8. doi: 10.1155/2022/2533413

[37] Vaszkun B Szakács KM. Looking for student success factors outside of the educators’ scope: the effect of digital literacy, personal skills, and learning habits and conditions on self-evaluated online learning effectiveness in management education. Int J Manag Educ. (2025) 23:101188. doi: 10.1016/j.ijme.2025.101188

[38] Bahri A Jamaluddin AB Arifin AN Saparuddin. Students’ and teachers’ digital literacy skill: a comparative study between schools, classes, and gender in urban and rural areas. Int J Sci Res. (2022) 11:184–91. doi: 10.21275/SR22130101519

[39] Aydınlar A Mavi A Kütükçü E Elgün Kırımlı E Alış D Akın A . Awareness and level of digital literacy among students receiving health- based education. BMC Med Educ. (2024) 24:38. doi: 10.1186/s12909-024-05025-w PMC10773083 38191385

[40] Alharbi A Alkubati SA Albaqawi H Ali AZ Hamed LA Mohammed S . Mediating roles of psychological empowerment and self-efficacy in the relationship between leadership and clinical decision-making among nursing students: a path-analysis approach. Nurse Educ Today. (2026) 161:107022. doi: 10.1016/j.nedt.2026.107022, 41687335

[41] Gause G Mokgaola IO Rakhudu MA. Technology usage for teaching and learning in nursing education: an integrative review. Curationis. (2022) 45:a2261. doi: 10.4102/curationis.v45i1.2261, 35792609 PMC9257720

[42] Zainal NH Islam MA Rasudin NS Mamat Z Hanis TM. Critical thinking and clinical decision making among registered nurses in clinical practice: a systematic review and meta-analysis. Nurs Rep. (2025) 15:175. doi: 10.3390/nursrep15050175, 40423210 PMC12113899

[43] Raghunathan K McKenna L Peddle M. Baseline evaluation of nursing students’ informatics competency for digital health practice: a descriptive exploratory study. Digit Health. (2023) 9:20552076231179051. doi: 10.1177/20552076231179051 PMC10236246 37274371

[44] Aboobaker N Zakkariya KA. Influence of digital learning orientation and readiness for change on innovative work behaviour: reflections from the higher education sector. Develop Learn Organ. (2020) 34:25–8. doi: 10.1108/DLO-08-2019-0191, 35579975

[45] Han J. The mediating effect of problem solving ability on the relationship between digital literacy and clinical performance ability in nursing students. Architec Image Stud. (2026) 7:137–42. doi: 10.62754/ais.v7i1.648

[46] Lee J-Y Ha Y Kim M. Effects of digital literacy and communication competency on clinical competency in nursing students. Korean Assoc Learn Center Curric Instruct. (2024) 24:499–509. doi: 10.22251/jlcci.2024.24.5.499

[47] Cunha LDM Ventura F Pestana-Santos M Mota M Lomba L Santos MR. Decision support strategies for bedside nursing clinical reasoning: a scoping review. Int J Nurs Stud Adv. (2025) 9:100393. doi: 10.1016/j.ijnsa.2025.100393, 40800610 PMC12341538

[48] Saban M Dubovi I. A comparative vignette study: evaluating the potential role of a generative AI model in enhancing clinical decision-making in nursing. J Adv Nurs. (2024) 81:7489–99. doi: 10.1111/jan.16101, 38366690 PMC12535322

